# Impacts of government supervision on hospitalization costs for inpatients with COPD: An interrupted time series study

**DOI:** 10.1097/MD.0000000000018977

**Published:** 2020-01-31

**Authors:** Peiyi Li, Zhanqi Duan, Ziwu Zhang, Yunzhen He, Weimin Li, Jin Wen

**Affiliations:** a Institute of Hospital Management, West China Hospital, Sichuan University; b Department of Statistics and Survey, Health and Family Planning Commission of Sichuan Province; c Department of Health Economics, School of Public Health, Fudan University, Shanghai; d Department of Respiratory Medicine, West China Hospital of Sichuan University, Chengdu, China.

**Keywords:** chronic obstructive pulmonary disease, government supervision, medical costs, medical misconduct, Zero-Makeup Drug Policy

## Abstract

To address the remaining medical misconducts after the zero-makeup drug policy (ZMDP), e.g., over-examinations, China has given the priority to government supervision on medical institutions. This study evaluated the effect of government supervision on medical costs among inpatients with chronic obstructive pulmonary disease (COPD) in Sichuan province, the first province in China where the medical supervision was conducted.

A linear interrupted time series (ITS) model was employed to analyze data about 72,113 inpatients from 32 hospitals. Monthly average medicine costs, diagnostic costs, and medical services costs, nursing costs from January 2015 to June 2018 were analyzed, respectively.

The average hospitalization costs fell with a monthly trend of 42.90Yuan before the implementation of supervision (*P* < .001), and the declining trend remained with the more dramatic rate (−158.70Yuan, *P* < .001) after the government audit carried out. For western medicine costs, the monthly decreasing trend remained after the implementation of supervision (−66.44Yuan, *P* < .001); meanwhile, the monthly upward trend was changed into a downtrend trend for traditional Chinese medicine costs (−11.80Yuan, *P* *=* .009). Additionally, the increasing monthly trend in average diagnostics costs disappeared after government supervision, and was inversed to an insignificant decreasing trend at the rate of 26.18Yuan per month. Moreover, the previous upward trends were changed into downward trends for both medical service costs and nursing costs (*P* = .056, −44.71Yuan; *P* = .007, −11.17Yuan, respectively) after the supervision carried out.

Our findings reveal that government supervision in Sichuan province was applicable to curb the growth of medical costs for inpatients with COPD, which may reflect its role in restraining physicians’ compensating behaviors after the ZMDP. The government medical supervision holds promise to dismiss medical misconducts in Sichuan province, the experience of which may offer implications for other regions of China as well as other low- and middle-income countries.

## 1 Introduction

The transition from the planned economy era to the market-oriented economic era in the 1980s has led to a reduction in revenues among China's health providers.^[1]^ To compensate for the reduction, health providers were allowed to charge an extra 15% profit margin based on drugs procurement prices as markup.^[2]^ Thus, physicians were encouraged to over-prescribe drugs, even some of which may be unnecessary for patients.^[3,4]^ Complaints about unaffordable healthcare expenses in public hospitals, the main providers of healthcare service in China, have gradually increased.^[5,6]^ To restrain over-prescription and control health costs, the Ministry of Health of Chinese government proposed the Zero-Makeup Drug Policy (ZMDP) in 2009, which canceled the 15% drug markup. The initiative indeed achieved its goal of eliminating over-prescribing immediately.^[7]^ While the increase of health cost remained since physicians turned to other lucrative services,^[8]^ such as prescribing superfluous examinations and tests.^[9,10]^ In addition, citizens were more likely to pursue unnecessary prescriptions and tests since the reimbursement ratio of medical insurance have increased drastically in recent years, especially for retirees.^[11]^ Consequently, these proceeding misconducts have continuously contributed to the waste of scarce medical resources and unbearable financial burden for Chinese.^[12,13]^

In order to deal with these public concerns about medical misconducts and over-treatments, the Chinese government proposed that supervising medical institutions via information technology might be an effective strategy to address these problems.^[14–16]^ As the pioneer in China, the Department of Health in Sichuan Province established the first electronic supervising platform with the aim of conducting full-process monitoring of medical behaviors and costs in December 2016.^[17]^ The platform has been successfully operated and connected with all medical institutions in Sichuan province since July 2017. Thirty measures, including unreasonable prescriptions, unreasonable tests, and average fees for each prescription, were monitored to analyze medical behaviors. As the first governmental initiative to supervise medical institutions electronically, it remains, however, unclear whether the implementation has achieved its intended goal of curbing health costs, such as diagnostic costs.

Chronic obstructive pulmonary disease (COPD), characterized by a progressive deterioration of lung function, is a major public health concern in China with its high prevalence, mortality, and fiscal costs.^[18,19]^ Patients with COPD are usually accompanied by both mental and physical comorbidities such as dystrophy and heart failure,^[20]^ and require multiple medicines and radiographic examinations throughout their stay in hospitals. Therefore, our study aimed to evaluate the effectiveness of first China's medical supervision program on the hospitalization costs for inpatients with COPD.

## 2 Materials and methods

### 2.1 Study setting

The present study included data about 72,113 insured inpatients in Sichuan province, which is located in Southwestern China, with land area of over 48.6 million square kilometers and 83.41 million population.^[21]^ The study sample consisted of inpatients from 32 medical institutions, which were representatives of medical institutions in Southwestern China for the following reasons. First, we included hierarchical (tertiary, secondary, and ungraded hospitals) and multifunctional (general, specialized, and traditional Chinese medical hospitals) medical centers. In addition, we included hospitals of different hospital bed capacity and the leading hospitals in the regional medical alliance. Table 1 presents the characteristics of the 32 hospitals in detail.

**Table 1:**
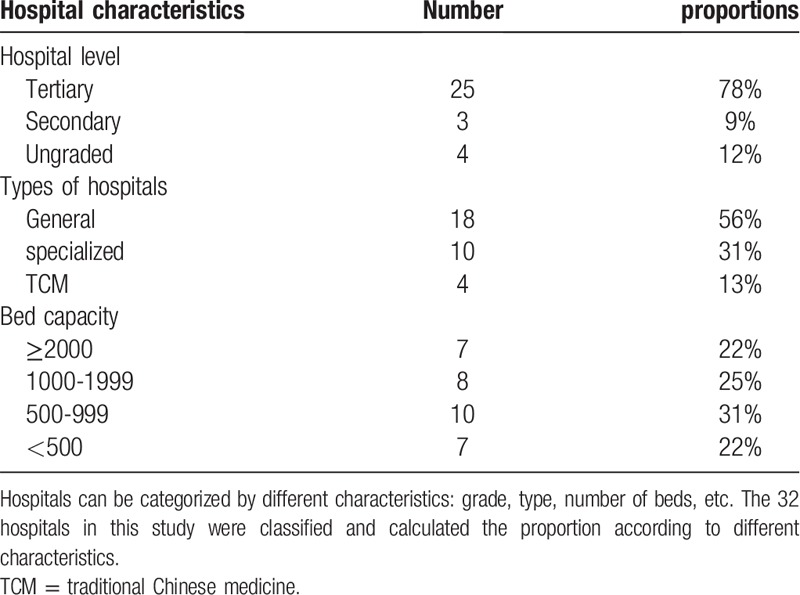
Characteristics of 32 representative hospitals.

### 2.2 Specific policy intervention

The supervising program was carried out by the Department of Health in Sichuan Province with the tiered context of medical institutions, physicians and medical behaviors. First, the regulatory indicators were defined in several ways to ensure compliance with professional standards and clinical guidelines. Second, the supervising platform was operated to automatically audit those indicators and analyze whether there were any medical misconducts based on horizontal comparison between various institutions and medics, as well as vertical comparison of individual behaviors. If any abnormal behaviors were identified, the inspectors would visit both the administrator of the medical institutions and the physician himself or herself. Then the Department of Health would assess whether the abnormal behaviors are medical misconduct or reasonable ones by reviewing documents relative to the patient, such as treatment protocols and patient files. If the unusual behavior was identified as medical misconduct, then the organization and physician must take measures to dismiss their malpractices, which should be reported to the Department of Health in Sichuan Province. The inspectors are given the right to take mandated steps if the organization does not comply and there is insufficient faith in the organization to eliminate the improper conducts in time. For instance, the inspectors may frequently visit the institution, release media announcements, and penalize the physician including prohibiting him/her, temporarily or even permanently from accepting new patients.

### 2.3 Outcome variables and data sources

In this study, the total hospitalization costs for inpatients with COPD were mainly incurred by prescription drugs, diagnostic tests, medical services and nursing care. Among these, the drugs were comprised of western medicine and traditional Chinese medicine (TCM). Our primary outcome was defined as the monthly average medical costs, which were calculated via dividing the total expenditures of each medical expenses by the number of inpatients per month. This was done to avoid the bias of results attributes to fluctuations in the number of inpatients in each month. The supervising policy was officially implemented in July 2017, with data in a total of 42 monthly time periods from January 2015 until June 2018 (30 months before and 12 months after the implementations) collected.

After approved by the Institutional Review Board of West China Hospital of Sichuan University, data were derived from electronic health records at the 32 hospitals with personal and hospital identification information removed for protecting privacy. Inpatients (1) that hospitalized less than 2 days or more than 60 days; and (2) experienced surgical treatments were excluded.

### 2.4 Statistical analysis

The ITS, regarded as the strongest and quasi-experimental approach, was used to evaluate the longitudinal effects of the supervision platform.^[22,23]^ The segmented regression analysis of ITS was employed to examine (1) how much an intervention changed an outcome of interest immediately and over time; and (2) whether factors other than the intervention could explain the change. The function of the model is specified as followed: 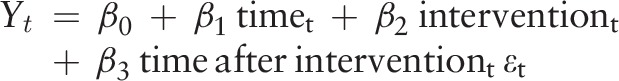


Here, *Y*_t_ is the outcomes variable in time t; time_t_ is a continuous variable counting the number of months at time t from the start of the observation period. In our study, the values of time_t_ range from 1(first period) to 42 (last period). The intervention_t_ is a binary variable coded 0 for the period before the intervention and 1 for after the intervention, which was implemented at month 31. And time after intervention_t_ is a continuous variable counting the number of months after the intervention at time t, which was set at 0 before the reform and sequentially numbers after the supervision. The *ε* represents the random variability not explained by the model at time t. For parameters, *β*_0_ estimates the baseline level of the outcome variable, while *β*_1_ captures the monthly change in outcome variable that occurs with each month before the intervention (i.e., baseline trend), *β*_2_ estimates the changes in the value of the outcome variable in the first month after the intervention (i.e., immediately effect of the intervention on outcome) and *β*_3_ estimates the monthly change in the trend after intervention (compared with the pre-intervention trend).

A full set of seasonal dummy variables was incorporated to minimize the seasonal fluctuations in our data. Our interrupted time series regression model was estimated by auto-regressive integrated moving average method (“ARIMA”), with the first-order autoregressive procedure (“AR (1)”) to account for auto-correlation.^[24]^ Lastly, Huber-White robust estimates of the standard errors were computed for each model parameters to control for heteroscedasticity.^[25]^ All data analyses were performed using Stata/SE 15.0 (Stata Corporation College Station, TX, USA).

## 3 Results

**Figure 1:**
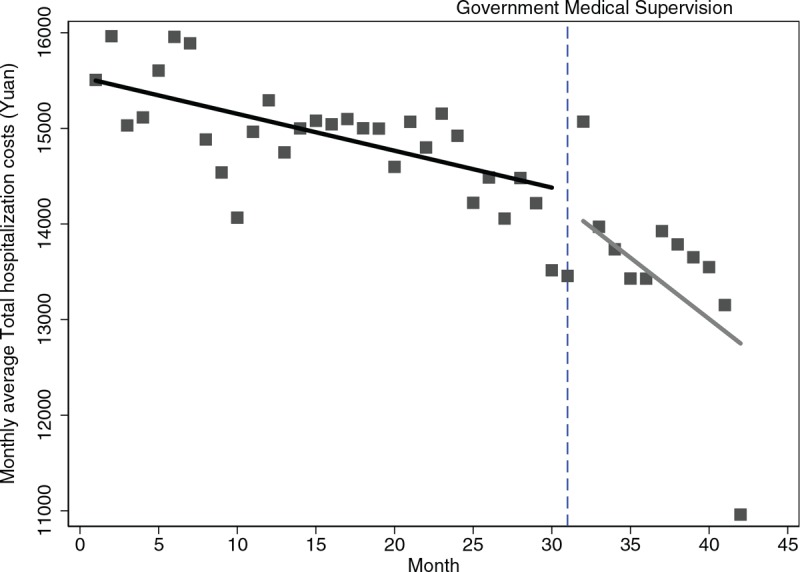
Trend in the monthly average Total hospitalization costs for 32 hospitals in Sichuan.

**Figure 2:**
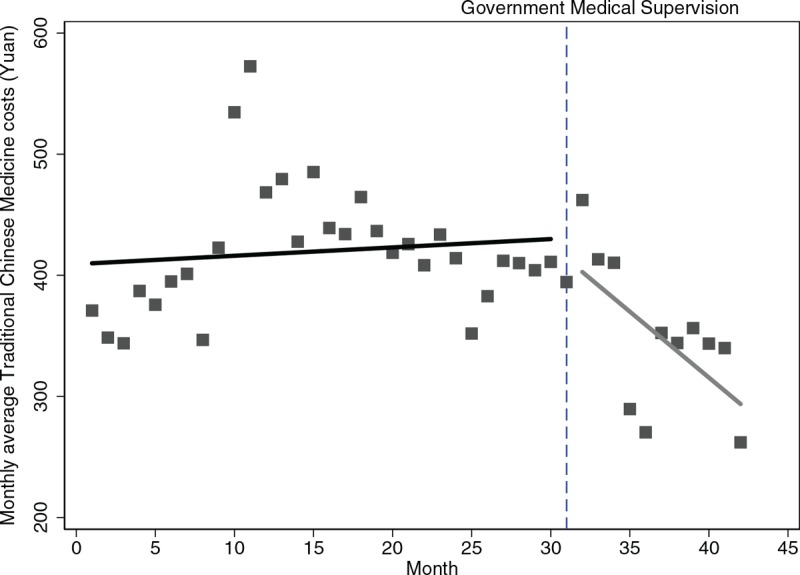
Trend in the monthly average TCM costs for 32 hospitals in Sichuan. TCM, Traditional Chinese medicine.

**Figure 3:**
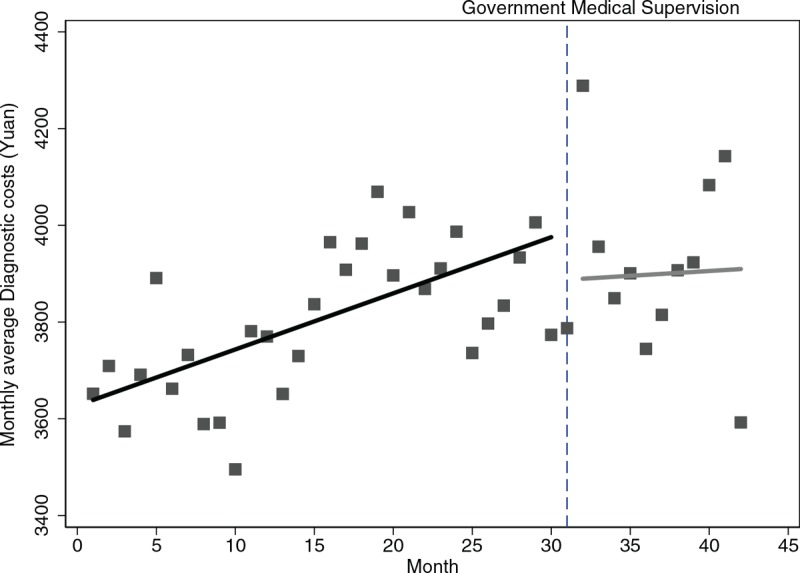
Trend in the monthly average Diagnostic costs for 32 hospitals in Sichuan.

Seventy two thousand one hundred thirteen inpatients with COPD across 42 months were enrolled in our study. Figure 1 delineates that monthly average inpatient costs saw a decrease of 42.90 Yuan before the implementation of government supervision (*P* < .001), whereas, it witnessed a sharper decrease of 158.70 Yuan in the following months (*P* < .001). The regression model was demonstrated as: 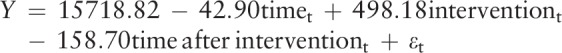


Table 2 further depicts the numerical details of level and trend changes in medical costs before and after the supervision. Specifically, the month-to-month decrease of western medicine costs continued with a statistically significant (*P* < .001). Figure 2 also depicted an intuitive display of monthly changes in average TCM costs, in which the upward trend before supervision was reversed to a downward trend by 11.80 Yuan (*P* = .009). It was worth mentioning that the previous monthly increasing rate of 10.24 Yuan of diagnostic disappeared and turned into a declining trend of 26.18Yuan per month following the supervision continued (Fig. 3), but insignificant (*P* = .17). In terms of medical service costs and nursing costs, both the monthly increased trends before the supervision persisted at the first month of supervision were changed into the declining trends with rates of 44.71Yuan and 11.17Yuan per month as supervision carried out (*P* = .056, *P* = .007 respectively).

**Table 2:**
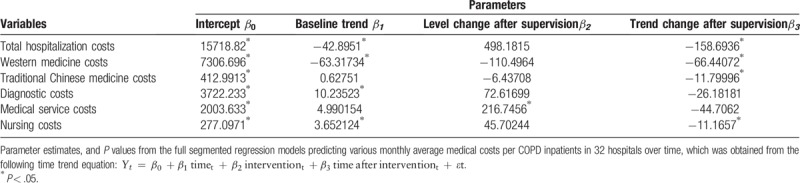
Estimated coefficients of segmented regression model for the monthly average medical costs in 32 hospitals before and after supervision (RMB,Yuan).

Furthermore, the proportion of total drug expenses accounted for the highest percentage of the total hospitalization costs (60%) in 2015, followed by diagnostic (26.3%) and medical service (13.1%). After 3 years, the ratio gradually changed respectively: drug (4.6% decrease), diagnostic (4.3% increase) and medical service (3.2% increase) considerably.

## 4 Discussion

This work represents the first step in understanding the realistic significance of government supervision in alleviating the financial burden for inpatients with COPD across China. Research suggests the implementation of the electronic supervision technology could be promising in low and middle-income countries (LMICs), which went through similar transitions of medical service model with China. Our findings contribute to the literature around governmental initiatives that hold the promise to promote healthcare reform.

Before the implementation of government supervision, we observed a remarkable decreasing trend in total hospitalization costs and western medicine costs which resulted from the launch of ZMDP,^[26]^ and the results were consistent with those in previous studies.^[27]^ However, our study demonstrated the average total hospitalization costs for inpatients with COPD were 15,609 Yuan before July 2017, equivalent with per capita annual income of rural people contemporary, which undoubtedly continued to pose a heavy economic burden in Chinese.^[28,29]^ What's worse, the magnitude of the reduction in total hospitalization costs was smaller than the western medicine costs due to the increasing diagnostic and nursing costs, which was also testified in another study, in which the declining effects of ZMDP on hospitalization costs had become weakened and turned into risen eventually.^[30]^ This was because physicians turned to prescribe other lucrative services to compensate for the reduction in drug kickbacks after the ZMDP and insufficient government financial subsidies.^[31,32]^ In our study, the TCM, which was not included in the list of ZMDP, has been excessively prescribed in order to increase revenues.^[33]^ And the diagnostic costs, accounted for the second-largest proportion of total hospitalization costs for inpatients with COPD, no doubt became the target of health providers to offset their loss in drug revenues. Nursing costs, which were easily overlooked because of it taking up the smallest proportion of total medical costs, also appeared to become one of the providers’ targets for compensating the reduction of drug rebates in our study.

Although the Chinese government has tried a series of initiatives to control the soaring health expenses since 2009, the people did not benefit that much due to the persistent existence of various medical misconducts,^[34,35]^ which motivated Chinese government to seek further methods to eradicate over-treatment and misconducts since 2016.^[36]^ Previous studies have demonstrated that clinical supervision of health professionals could not only improve quality of care,^[37–40]^ but also controlled the medical expenses.^[41]^ Therefore, the medical supervision platform in Sichuan province was established to real-time audit medical behaviors, especially over-treatments evoked by improper intends. As the supervision program was implemented, declining trends for almost all medical costs were observed, which suggested the Sichuan supervision model has yielded initial success in decreasing the medical expenses of COPD. The remarkable impacts of the first supervision platform in China may ascribe to constraints of economy and reputation. The inspectors would interview the dean of the hospital and the director of the department once unreasonable medical behaviors were identified via the supervision platform, and physicians with serious medical misconducts would be deprived of medical qualification and lose their job. Meanwhile, the monitoring results would also be considered while evaluating the hospitals and personal, which directly affect the income and reputation of institutions and physicians. Health care professionals started to realize that inappropriate behaviors evoked by financial incentives may not only lead to punishment but also affect their reputation and career.

Reforming Chinese public hospitals is particularly difficult for the balance of physicians’ income and public welfare.^[42,43]^ In the past, researchers paid considerable attention to internal incentives for medical staff to over-treatment, but have not given enough attention to the external role of government.^[44]^ The supervision platform of Sichuan demonstrates that the government monitor could coordinate with other initiatives of China medical reform and serve as an effective tool to improve the performance of public hospitals and benefit the people. It was consistent with Brickley study that suggested both internal and external management have to be aligned to influence clinical practices directly.^[45]^ The Sichuan supervision model aligned with other policies could boost the success of health care reform in LMICs which are currently going through similar transitions of the medical service model.

### 4.1 Strength and limitations

This study was conducted in Sichuan province with a large-size study sample to represent the population in Southwestern China. We employed segmented regression analysis of ITS, which is a robust modeling method to measure the dynamic changes following intervention when randomization or identification of a control group was not applicable.^[46]^

Despite the strength, our study has several limitations. First, we were unable to determine the changes in outpatients with COPD, and that the overall picture regarding the overall costs for this disease could be increased, but also may decrease using the investigated intervention. Further research is needed to investigate the behavioral responses to the supervisions that led to the whole patients with COPD. Secondly, in the present study, we only included the diseases of COPD, thereby one should be cautious to generalize findings to patients with other diseases. Moreover, the impact of supervision on the outcome of treatment was not investigated. Further studies should examine changes in healthcare quality and safety in order to evaluate the government supervision model comprehensively.

## 5 Conclusions

The government supervision of Sichuan province had achieved its objectives of alleviating the economic burden and curbing misconducts, which were compensations for western medicine rebates after ZMDP. As China is planning to launch the deep round of medical reform in the next decades, the supervision platform of Sichuan province helps to set a demonstration of using information technology to provide early warning and timely correction of medical misconducts, which is essential for the success of China healthcare reform.

## Acknowledgments

We also thanks Dr. Xun Yang, Dr. Xiaolin Guo and Dr. Xu Han for the contribution of data sources. The authors would like to sincerely acknowledge Dr. Xuexin Yu and Xingyou Wang for their assistance in making this a better article.

## Author contributions

JW was the principal designer of the proposed study. PY-L led the development of this manuscript and approved all changes. WM-L contributed to the research design and critically revise the manuscript. ZQ-D, ZW-Z were involved in data curations and revised the manuscript for important intellectual content. YZ-H was closely involved in data analysis and interpretation. All authors read and approved the final manuscript.
